# Analysis of Risk Factors and Consequences of Consecutive Proximal Femur Fractures in Elderly Patients

**DOI:** 10.7759/cureus.18527

**Published:** 2021-10-06

**Authors:** Suranga Gurusinghe, Devaraj M Navaratnam, Konara Weerasinghe, Girish Gopinath, Chika Uzoigwe, Theophilus Joachim

**Affiliations:** 1 Trauma and Orthopedics, Pilgrim Hospital, Boston, GBR

**Keywords:** risk factors, contralateral fracture, dynamic hip screw fixation, hip hemiarthroplasty, proximal femur fractures

## Abstract

Background

Proximal femur fracture (PFF) carries significant morbidity, mortality, and cost implications to the health system. Subsequent contralateral fracture further decreases patient performance and increases the healthcare burden. This study aimed to identify and evaluate potential risk factors for consecutive PFF.

Methodology

Pilgrim Hospital PFF database from 2012 to 2019 was retrospectively analyzed. Patients over 60 years with low-energy fractures were included. Pathological and atypical fractures and polytrauma were excluded.

Results

There were 114 patients (4.18%) with contralateral hip fractures out of a total of 2727 PFF patients; 80% were females. The mean age was 82 years for the first hip fracture and 85 years for the second. The average time interval between fractures was 36 months. The fracture pattern was the same on both sides in 74.3% of patients (P<0.0001). Out of 53 patients with cemented hip hemiarthroplasty (CHH) on one side, 31 patients (59%) had a second CHH for the contralateral side. Likewise, out of 48 patients who had dynamic hip screw fixation during the first admission, 33 patients (69%) had the same procedure on the contralateral side too.

During the two consecutive admissions, the length of hospital stay was not significantly different (P=0.30), median American Society of Anesthesiologists (ASA) grades were 3, hyponatremia increased from 25% to 29% (P=0.5), mean decline in abbreviated mental test score (AMTS) was 0.4, deterioration of Clinical Frailty Score and Charlson morbidity index were from 4.5 to 5.9 (P<0.0001), and from 5.4 to 6.1, respectively, and institutional residency was increased from 23 to 46 (P>0.0014).

Conclusion

The similarity of fracture pattern bilaterally requiring similar surgical procedures is comparable with other literature. Even though there is minimal or no change in the ASA, AMTS, and hospital stay between the two admissions, there is a significant decline in clinical frailty, mobility status, and an increase in residential dependency following a subsequent fracture. Our findings demonstrate the importance of emphasizing secondary preventive measures to prevent a consecutive fracture.

## Introduction

Longer life expectancy and increased elderly population density result in 65,000 proximal femur fracture (PFF) admissions to the United Kingdom hospitals annually. This is expected to increase further every year. Statistically, this is comparable to other western countries. This is a major public health concern globally [1]. PFF has a devastating impact not only on the older patients but on their families as well. Studies have shown a 30-day mortality rate of 9%, and a one-year mortality rate of 32% [2].

Factors that can contribute to the fall include generalized frailty and lack of physical strength. Indirect factors are neurological impairments, cognitive impairments, other comorbidities affecting locomotion, and declining musculoskeletal health. The risk of osteoporotic fracture in a lifetime is 30%. Most osteoporotic fractures do not lead to life-changing consequences as much as PFF does. Index PFF leads to a gradual decline in bone mineral density (BMD) of the contralateral proximal femur (CPF). However, this does not directly explain the lowering mechanical strength of the unaffected hip, significantly [3]. Results of comparative structural analysis of the CPF and age-matched control groups clearly show a greater loss of geometric strength of CPF, especially in narrower regions of the neck and intertrochanteric region [4].

Risk of a second hip fracture following an index hip fracture range from 2% to 13% in various studies, with 50% occurring within the first two years [2,5]. Furthermore, postoperative complications, prolonged hospital stays, reduction in mobility status, and significantly higher mortality rates are seen more frequently with contralateral fractures [6]. 

These denominators are negatively affecting individual performance and dependency. This population is overburdening the healthcare system and social services. Evidence for proximal femoral fractures are easily accessible. However, there is very limited consensus for a second PFF. Compared to other studies, we used a more pragmatic approach and broadly assessed risk factors as well as consequences of consecutive PFF. 

Our study, which covered a longer duration and larger cohort, yields stronger inferences. The primary objective of our study was to identify patterns, risk factors, and consequences of developing a second PFF.

## Materials and methods

The hip fracture database of Pilgrim Hospital was retrospectively analyzed from January 2012 to December 2019. The patients above 60 years of age with low-velocity PFF at intra-capsular, intertrochanteric, and subtrochanteric levels were included in this study. Polytrauma, pathological fractures, atypical fractures, and those with ipsilateral femoral shaft fractures were excluded from the study. 

Those PFF patients who subsequently suffered a contralateral low-energy proximal femur fracture were identified. Radiographs were independently observed by two separate clinicians to classify the fracture as per the AO system. Electronic discharge letters and in-patient investigations were accessed to obtain demographic and biochemical parameters. Medical comorbidities at index and second interventions were recorded. Charlson morbidity index and Clinical Frailty Scores were calculated for each admission. Mobilization status was given a numerical value to facilitate analysis: 0, 1, 2, 3, and 4, respectively, for an independent walker, walking with one stick, walking with a frame or two sticks, wheel-chair dependant, and bed bound. This was recorded separately for both admissions. The database and electronic records were used to identify the mortalities.

This study was conducted in full compliance with the ethical guidance for medical and human subjects laid down in the Declaration of Helsinki. It was also registered as a quality improvement project with the local clinical governance department. This was to identify any risk factors that the orthopedic department or the orthogeriatricians would be able to address.

## Results

Out of the 2727 recorded hip fracture patients during the seven-year period from 2012 to 2019, we had 114 (4.18% patients) with contralateral hip fractures. Mean ages for the first and contralateral fractures were 82 years and 85 years, respectively. Eighty percent of the cohort were female (91 females and 23 males). The average time interval between fractures was 36 months. Slightly over 50% <53%, 57/108> suffered the second proximal fracture in under two years (Tables 1, 2).

**Table 1:** First fracture type by treatment.

Procedures	Extra-capsular fractures	Intra-capsular fractures
Cemented hemiarthroplasties	0	53
Uncemented hemiarthroplasties	0	7
Cephalomedullary nail fixations	2	0
Cannulated screw fixations	0	2
Dynamic hip screw fixations	39	8
Total hip replacements	0	2

**Table 2:** Cumulative incidence of consecutive PFF. PFF: proximal femur fracture

Year since index procedure	Cumulative number of contralateral PFF	Cumulative percentage of PFF
1st year	22	20%
2nd year	57	53%
3rd year	71	66%
4th year	91	84%

The fracture pattern was the same on both sides in 74.3% of patients (P<0.0001). Out of 53 patients who underwent CHH of the hip as the index procedure, 31 (59%) had the same intervention on the contralateral side (Tables 3, 4). Forty-six patients had dynamic hip screw fixation for a first fracture and 32 (69%) of those underwent the same on the opposite side (Tables 3, 4). Hyponatremia (< 133 mEq/L) was noted in 26 (25%) out of 104 patients at the index admission and in 33 (29%) out of 113 patients during the second admission. This was found statistically not significant (P=0.5) (Table 3). The median ASA grade for both admissions was 3. The mean decline in abbreviated mental test score (AMTS) from index to second event was 0.4. However, 80/104 (77%) retained the same AMTS score of 9. The mean decline in Charlson morbidity index between two consecutive admissions was 0.6 (P<0.0001). The morbidity index was unchanged in 14% (15/104) of the cohort (Table 3).

**Table 3:** Demographic features, risk factors, and consequences for the first and second hip fractures. PFF: proximal femur fracture; AMTS: abbreviated mental test score; ASA: American Society of Anesthesiologists

Variables	First PFF	Second PFF
Mean age (SD)	82.6 (9.0)	85.1 (8.3)
Median (quartiles)	84 (78-89)	87 (80-91)
Fracture type		
Extra-capsular fractures	41	50
Intra-capsular fractures	72	62
Sub-trochanteric fractures	0	2
Surgical treatment		
Dynamic hip screw (DHS) fixations	46	52
DHS+ de rotation screw (DRS)	1	2
Cannulated screw fixations	2	1
Cephalo-medullary nail (CMN) fixations	2	2
Cemented hemiarthroplasties	53	46
Uncemented hemiarthroplasties	7	10
Total hip replacements (THR)	2	1
AMTS (median, quartiles)	9 (4-10)	9 (2-10)
ASA	3 (2-3)	3 (3-3)
Charlson morbidity index	5 (4-7)	6 (5-7)
Mobility index	1 (0-2)	2 (2-3)
Clinical frailty index	5 (3-6)	6 (5-7)
Blood sodium	137 (134-140)	138 (134-140)
Blood potassium	4 (3.6-4.4)	4.1 (3.8-4.4)
Length of hospital stay (median, quartiles)	11 (8-16)	12 (8-20)
Length of hospital stay by fracture type (median)		
Extra capsular fractures	10.0	12.5
Intra capsular fractures	12.5	12.0
Sub trochanteric fractures	-	14.0
Infections		
Respiratory tract infections	4	7
Urinary tract infections	24	21
Sepsis of unknown origin	0	3
None	85	82
Discharged location		
Home	90	67
Nursing home	9	17
Residential home	14	29
Deceased (N, %)		4 (2.6%)

**Table 4:** Second fracture type by treatment.

Procedures	Extra-capsular fractures	Intra-capsular fractures	Sub trochanteric fracture
Cemented hemiarthroplasties	0	46	0
Uncemented hemiarthroplasties	0	10	0
Cephalomedullary nail fixations	0	0	2
Cannulated screw fixations	0	1	0
Dynamic hip screw fixations	49	5	0
Total hip replacements	0	1	0

There was a mean drop of 1.4 in the Clinical Frailty Score (CFS) from 4.5 to 5.9 (P<0.0001). The mean drop in mobility status was by a factor of 1 (P<0.00001). There was no significant difference in length of hospital stay (P=0.3). Twenty-three out of 114 patients were from a residential home at the index admission. For the second admission, double that number (46) came in from either resident or nursing homes (P>0.0014).

## Discussion

This study shows that 4% of our cohort had subsequent contralateral PFF. This is in line with other literature of 2-13% prevalence where 50% of the contralateral fracture occurred within the first two years of the index fracture. Among the common causes is an increase in the risk of falling following index trauma, longer rehabilitation process following surgical intervention, slow learning curve of walking re-training, and worsening of other age-related conditions, especially those involving the musculoskeletal system [7].

The average life expectancy is 81 years (female 83 and male 79) in the United Kingdom. The mean age at presentation of the first and second PFF were 82 and 85 years, respectively. Our cohort of patients with contralateral fracture has survived longer considering the average life expectancy and mortality rate after PFF. We postulate that our patients are healthier and more resilient to surgical intervention due to their rural advantage, as described by Sanders et al. [8].

Eighty percent of patients in our study were female. Males have higher bone mass when compared to females. However, when males are much older, their bones exhibit an exponential loss in bone mass compared to females [9]. Hence, we would expect to see more male PFFs. Males however have a lower life expectancy (79 compared to 83) and therefore are less likely to survive long to sustain a subsequent contralateral neck of femur fracture, when compared to the female counterpart.

The similarity of the anatomical location of two consecutive PFFs in this series was comparable with other literature [10-12]. There are different theories postulated to explain this phenomenon such as alteration of gait pattern, reduction in BMD, and changes in the geometry of the second proximal femur.

In terms of risk factors, a decline in cognitive status and a resultant lack of awareness of surroundings are the previously known factors contributing to falls. However, our data revealed only a 0.4% decline in AMTS between the two consecutive PFFs. Median AMTS was 9 and 77% retain their AMTS following the contralateral fracture. CMI deteriorated from 5 to 6, representing increase morbidity during the two consecutive PFFs.

The median ASA score was 3 (severe systemic disease) on both occasions. There is a wide gap between the scoring of ASA 3 and 4 (severe systemic disease with the constant threat to life). This could have caused some selection bias in categorizing the score due to variability among individual clinicians, depending on the level of their experience and perception of the importance of the score.

Mobility status of patients dropped by one level with each PFF (i.e., independent walker to stick dependant, single stick dependant to dual-stick or frame, frame dependant to wheelchair, and wheelchair-dependent patients to bed-bound status). CFS also demonstrated a decline comparable to mobility index. CFS though represents a combination of mobility, medical comorbidities, and cognitive status.

Infection as a causative factor for the second PFF has been recently published [13]. It can cause an alteration in the cognitive status and balance, thus leading to falls. In our study, infection markers were positive in 29/113 index and 32/113 second admissions (D1). It would seem that infection is thus a risk factor for neck or femur fracture in both admissions. However, this was not deemed statistically significant between the two PFF groups.

Similarly, hyponatremia was present on both consecutive admissions in about 30% of the cohort. The chronically low sodium level is known to cause osteoporosis. Chronic, acute on chronic, and acute electrolyte imbalance could lead to alterations in coordination and balance, and eventually to an increased risk of falls [14]. However, impaired electrolyte balance appeared to have no significant impact on second PFF in our cohort.

Following the index procedure, 80% of patients were discharged back to their own homes. This fell to 60% in the subsequent admission. This demonstrates a stepwise deterioration and progressively increasing dependence associated with a repeat admission. The number of patients discharged back to institutions (residential or nursing homes) doubled during subsequent admissions compared to index admissions (20% at index, 40% in subsequent admissions). One would expect the length of hospital stay to be significantly higher for the second admission compared to the first. However, when we observed hospital stay in isolation, it is noted to have extended only by a single day (11-12 days).

Pre-operatively, independent people living in their own homes who were still marginally independent were discharged back to their usual place of abode. When patients became dependent, the discharge process took longer in order to facilitate a place of stay with an appropriate level of care, due to the rising demand for social care. On the other hand, vulnerable and institutionalized patients who were already in a suitable place of stay had a shorter course of in-patient stay which explained the inverse correlation between the place of residence (nursing home/residential) and duration of hospital stay. The lowest 30-day mortality report by National Hip Fracture Database (NHFD) is 6.1%. In our series, the 30-day mortality was very low at 2.6% (4/114) for consecutive PFF. This may be due to the fact that our cohort of patients was generally healthier and more resilient.

As a part of the preventive program in the National Institute for Clinical Excellence (NICE) guideline for PFF, it is recommended to assess mental status, bone protection, and fall risk. We achieved beyond 90% compliance on assessment of the above three categories. However, the actual anti-resorptive prescription was low in line with the rest of the world (Table 5). Poor understanding of the gravity of the situation by primary care providers, discontinuation of the drug due to adverse effects, and poor compliance of patients on oral treatment were thought to be the reasons for this poor anti-resorptive drug intake [15].

**Table 5:** Osteoporosis treatment.

Medication	Numbers of subjects
Alendronate	62
Denosumab	1
Ibandronate	3
Risedronate	1
Strontium	2
Unknown	1
None	44

Passive preventive measures such as hip protectors are more appropriate for cognitively impaired patients, patients with risk of falls, and poor mobility. Furthermore, regular physiotherapy assessment and provision of the most appropriate walking aids or custom-made walking aids are more pragmatic solutions. Sports medicine studies have revealed the importance of coordinated skills and muscle rehabilitation exercise as a more active method of engagement of elderly people for fracture prevention, 50% reduction of falls has shown with residential hazard reduction and individually tailored exercise programs even in mildly demented patients [15].

We suggest that healthcare professionals should focus on reducing the incidence of subsequent PFF as this causes a significant deterioration in the independent status of a patient. It impacts greatly on the family and poses enormous financial repercussions on health and social care. Active and passive fracture prevention measures should be formulated and aggressively incorporated into guidelines of care for the older population with an intention to minimize this risk of fall. 

This is study does have some limitations. The sample size of the study was small and limited to one hospital of the trust. Furthermore, there could be selective bias when categorizing ASA grades among clinicians hence the variation between the two groups. 

## Conclusions

The similarity of fracture pattern bilaterally requiring similar surgical procedures is comparable with other literature. Even though there is minimal or no change in the ASA, AMTS, and hospital stay between the two admissions, there is a significant decline in clinical frailty, mobility status, and an increase in residential dependency following a subsequent fracture. Our findings demonstrate the importance of emphasizing preventive measures.

We suggest active and passive fracture prevention measures should be formulated and aggressively incorporated into guidelines of care for the older population with an intention to minimize this risk of fall.
